# Deep-blur: Blind identification and deblurring with convolutional neural networks

**DOI:** 10.1017/S2633903X24000096

**Published:** 2024-11-15

**Authors:** Valentin Debarnot, Pierre Weiss

**Affiliations:** 1 Departement Mathematics and computer science, Basel University, Basel, Switzerland; 2 Institut de Recherche en Informatique de Toulouse (IRIT), CNRS & Université de Toulouse, Toulouse, France; 3 Centre de Biologie Intégrative (CBI), Laboratoire de biologie Moléculaire, Cellulaire et du Développement (MCD), CNRS & Université de Toulouse, Toulouse, France

**Keywords:** blind deblurring, deep learning, identification network, spatially variant blur, unrolled network

## Abstract

We propose a neural network architecture and a training procedure to estimate blurring operators and deblur images from a single degraded image. Our key assumption is that the forward operators can be parameterized by a low-dimensional vector. The models we consider include a description of the point spread function with Zernike polynomials in the pupil plane or product-convolution expansions, which incorporate space-varying operators. Numerical experiments show that the proposed method can accurately and robustly recover the blur parameters even for large noise levels. For a convolution model, the average signal-to-noise ratio of the recovered point spread function ranges from 13 dB in the noiseless regime to 8 dB in the high-noise regime. In comparison, the tested alternatives yield negative values. This operator estimate can then be used as an input for an unrolled neural network to deblur the image. Quantitative experiments on synthetic data demonstrate that this method outperforms other commonly used methods both perceptually and in terms of SSIM. The algorithm can process a 512 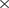 512 image under a second on a consumer graphics card and does not require any human interaction once the operator parameterization has been set up.^1^

## Impact Statement

The prospect of restoring blurred images with a wave of the digital wand is undeniably seductive in microscopy. However, the reality currently appears less satisfying, as handcrafted algorithms often offer only minimal gains at the price of long parameter tuning.

In this article, we combine physical models of the blur and artificial intelligence to design an interpretable blind deblurring method. A first neural network is trained to estimate the point spread function of the optical system, while a second network leverages this estimate to improve image quality. This approach provides a fully automated tool, capable of improving the image quality in seconds. The proposed methodology yields point spread function estimates with a quality that is superior by 10 dB to other popular methods, which also leads to better and more reliable deblurring results.

## 1. Introduction

Image deblurring and superresolution consist of recovering a sharp image 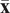 from its blurred and subsampled version 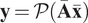 , where 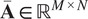 is a discretized linear integral operator describing the acquisition process, and 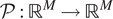 is some perturbation modeling noise, quantization, and saturation. It plays an important role in biomedical and astronomical imaging, where physical phenomena such as diffraction and turbulence strongly reduce the achievable resolution. It also received a constant attention in the field of computer vision, where moving or out-of-focus objects create artifacts. When the operator 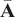 describing the optical system is available, this problem can be solved with mature variational inverse problem solvers^(^^16^^)^ or data-driven approaches.^(^^8^^)^

However, deriving a precise forward model requires specific calibration procedures, well-controlled imaging environments. and/or highly qualified staff. In addition, model mismatches result in distorted reconstructions. This can lead to dramatic performance loss, especially for superresolution applications.^(^^41^^,^^84^^)^

An alternative to a careful calibration step consists of solving the problem blindly: the forward model 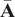 is estimated together with the sharp image 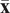 . Unfortunately, this blind inverse problem is highly degenerate. There is no hope to recover the sharp image without prior assumptions on 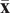 and 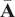 . For instance, assume that 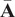 is a discrete convolution operator with some kernel 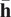 , that is, 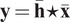 . Then, the couple 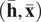 can be recovered only up to a large group of transformations.^(^^79^^)^ For instance, the identity and blurred image are a trivial solution, and the image and kernels can be shifted in opposite directions or scaled with inverse factors. Therefore, it is critical to introduce regularization terms both for the operator 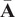 and the signal 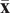 .

The main objective of this work is to design a blind inverse problem solver under the two assumptions below:

- The operator 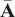 can be parameterized by a low-dimensional vector. In what follows, we let 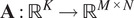 denote the operator mapping and we assume that 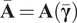 for some 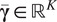 .
- The signal 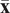 lives in a family 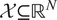 with some known distribution 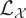 .

**Figure 1.**
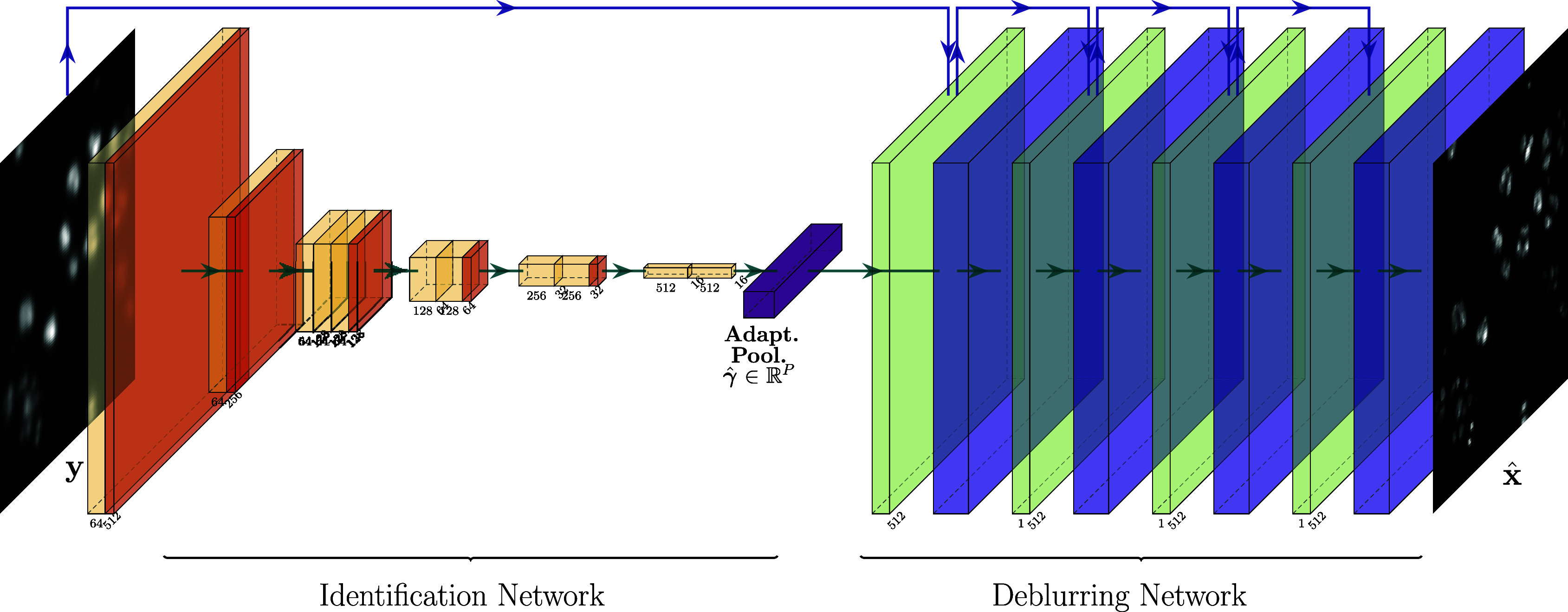
The deep-blur architecture. The first part of the network identifies the parameter 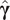 . In this article, we use a ResNet architecture. The estimated parameter 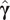 is given as an input of a second deblurring network. This one is an unrolled Douglas–Rachford algorithm. The yellow blocks are convolution layers with ReLU and batch normalization. The red ones are average pooling layers. The green ones are regularized inverse layers of the form 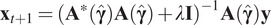 . The violet blocks are U-Net-like neural networks with weights learned to provide a sharp image 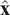 .

We propose a specific convolutional neural architecture and a training procedure to recover the couple 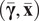 from the degraded data 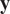 and the mapping 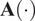 . A first network identifies the parameterization 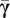 , while the second uses this parameterization to estimate the image 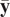 . This results in an efficient algorithm to sequentially estimate the blur operator and the sharp image 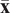 . The network architecture is shown on Figure 1. At a formal level, the work can be adapted to arbitrary inverse problems beyond image deblurring. We, however, showcase its efficiency only for challenging deblurring tasks involving convolutions but also more advanced space-varying operators.

### 1.1. Related works

Solving blind deblurring problems is a challenging task that started being studied in the 1970s.^(^^80^^)^ Fifty years later, it seems impossible to perform an exhaustive review of existing methods and the following description will be lacunary. We refer the interested reader to^(^^18^^)^ for a general overview of this field and to^(^^74^^)^ for a survey more focused on microscopy. The prevailing approach is to estimate the original signal and the blur operator by solving variational problems of the form:(1)

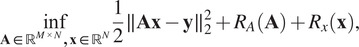

where 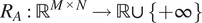 and 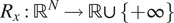 are regularization terms for the operator and the signal respectively. This problem arises when considering maximum a posteriori (MAP) estimators.^(^^50^^)^ It can be attacked with various types of alternating minimization procedures.^(^^11^^)^ Before the advent of data-driven approaches, the regularizers were carefully designed to target specific features. The point spread functions can be considered as sparse and compactly supported for motion deblurring.^(^^15^^,^^25^^,^^27^^,^^34^^,^^49^^,^^69^^,^^70^^,^^82^^)^ They are smooth for diffraction-limited systems^(^^17^^)^ and can also be parameterized with Zernike polynomials in the pupil plane.^(^^7^^,^^40^^,^^47^^,^^74^^,^^78^^,^^79^^)^ The images can sometimes be considered as sparse in microscopy and astronomical imaging^(^^31^^,^^60^^)^ or piecewise constant for natural images. The typical regularizer 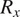 is then the total variation, or more advanced priors on the image gradient.^(^^9^^,^^15^^,^^17^^,^^68^^–^^70^^)^ Some authors also advocate for the use of priors on the image spectrum,^(^^38^^,^^86^^)^ which transform the blind deconvolution problem into a phase retrieval problem under ideal conditions.

The most recent variants of these approaches can provide excellent results (see e.g.,^(^^66^^,^^88^^)^). However, they strongly rely on the detection of specific features (points, edges, textures) which may be absent or inaccurate models of the typical image features. In addition, problem (1) or its derivatives is usually highly nonconvex, and the initialization must be chosen carefully to ensure local convergence to the right minimizer. As a result, these methods require a substantial know-how to be successfully applied to a specific field.

In the most recent years, machine learning approaches have emerged and now seem to outperform carefully handcrafted ones, at least under well-controlled conditions. These approaches can be divided into two categories. The first category concerns methods that directly estimate the reconstructed image from the observation.^(^^3^^,^^20^^,^^55^^–^^57^^,^^61^^,^^64^^,^^75^^)^ The second category contains approaches that produce an estimation of the blur operator. This estimate can then be used to deblur the original image. These approaches are specifically tuned for applications in computer vision^(^^14^^,^^39^^,^^51^^,^^75^^,^^81^^)^ (motion and out-of-focus blurs) or diffraction-limited systems.^(^^26^^,^^58^^,^^73^^,^^76^^,^^77^^,^^85^^)^ Our work rather falls in the second category.

In this list of references, a few authors propose ideas closely related to the ones developed hereafter. In particular,^(^^26^^,^^73^^,^^85^^)^ propose to estimate the pupil function of a microscope from images of point sources using neural networks. This idea is similar to the identification network in Figure 1. The two underlying assumptions are a space invariant system and the observation of a single point source. The idea closest to ours is from Shajkofci and Liebling.^(^^76^^,^^77^^)^ Therein, the authors estimate a decomposition of the point spread function from a single image using a low-dimensional parameterization such as a decomposition over Zernike polynomials. The spatial variations are then estimated by splitting the observation domain in patches where the blur is assumed locally invariant. The image can then be deblurred using a Richardson–Lucy algorithm based on the estimated operator.

### 1.2. Contributions

In this work, we propose to use a pair of convolutional neural networks to first estimate the operator parameterization 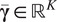 and then use this parameterization to estimate the sharp image 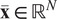 with a second convolutional neural network. The first network is the popular ResNet^(^^45^^)^ as in ^(^^77^^)^. The second network has the structure of an unrolled algorithm, which offers the advantage of adapting to the forward operator.^(^^1^^,^^2^^,^^59^^)^ We call the resulting algorithm deep-blur, see Figure 1. This work contains various original features:

- It includes space-varying blur operators that are accurately and efficiently encoded using product-convolution expansions as illustrated in ^(^^32^^,^^33^^)^. In particular, we show that this approach is compatible with the characterization of an optical system as a low-dimensional subspace of operators proposed in ^(^^28^^,^^29^^)^. Most approaches in the literature decompose the observation space into patches and treat each patch independently. In this work, we consider operators with an impulse response that varies continuously in the field of view.
- The resulting deblurring network is able to adapt to different forward models and to handle model mismatches naturally. This issue is an important concern for the use of model-based inverse problem solvers.^(^^6^^,^^36^^,^^65^^)^ As will be discussed later, our approach can be seen as an intermediate step between the plug-and-play algorithms^(^^83^^,^^87^^)^ and the unrolled algorithms.^(^^1^^)^
- We evaluate the efficiency, robustness, and stability of the proposed approach on various challenging problems, showing that the method is reliable and accurate.

The *PyTorch* implementation of our method is available on demand. We are currently integrating it into the DeepInv package.

## 2. Methods

In this article, we assume that the degraded signal 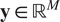 is generated according to the following equation:(2)

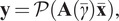

where 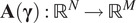 is a linear operator describing the optical system. It depends on an unknown parameter 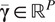 . The mapping 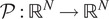 can model various deterministic or stochastic perturbations occurring in real systems such as additive white Gaussian noise, Poisson noise, and quantization. In this article, we will use a Poisson-Gaussian noise approximation detailed in ^(^^35^^)^. It is known to accurately model microscopes, except in the very low photon count regime. Other more complex models could be easily incorporated into the proposed framework at the learning stage. A critical aspect of this article is the parameterization of the forward operator 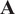 . We discuss this aspect below.

### 2.1. Modeling the blur operators

We consider both space-invariant and space-varying blur operators and linear or nonlinear parameterization.

#### 2.1.1. Linear parameterization

We may assume that 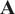 belongs to a subspace of operators.

#### 2.1.2. Convolution models and eigen-PSF bases

By far, the most widespread blurring model in imaging is based on convolution operators: the point spread function is identical whatever the position in space. This model is accurate for small fields of view, which are widespread in applications. Assuming that there is no subsampling in the model, we can set 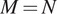 and 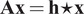 for some unknown convolution kernel 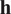 .

The convolution model strongly simplifies the blur identification problem since we are now looking for a vector of size 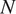 instead of a huge 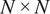 matrix. Yet, the blind deconvolution problem is known to suffer from many degeneracies and possesses a huge number of possible solutions, see for example ^(^^79^^)^. To further restrict the space of admissible operators and therefore improve the identifiability, we can expand the kernel 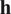 in an *eigen-PSF basis.* This leads to the following low-dimensional model.

**Model 2.1** (Convolution and eigen-PSFs). *We assume that*


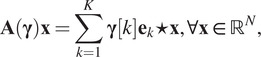

where 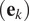 is an orthogonal family of convolution kernels called eigen-PSF basis.

**Figure 2.**
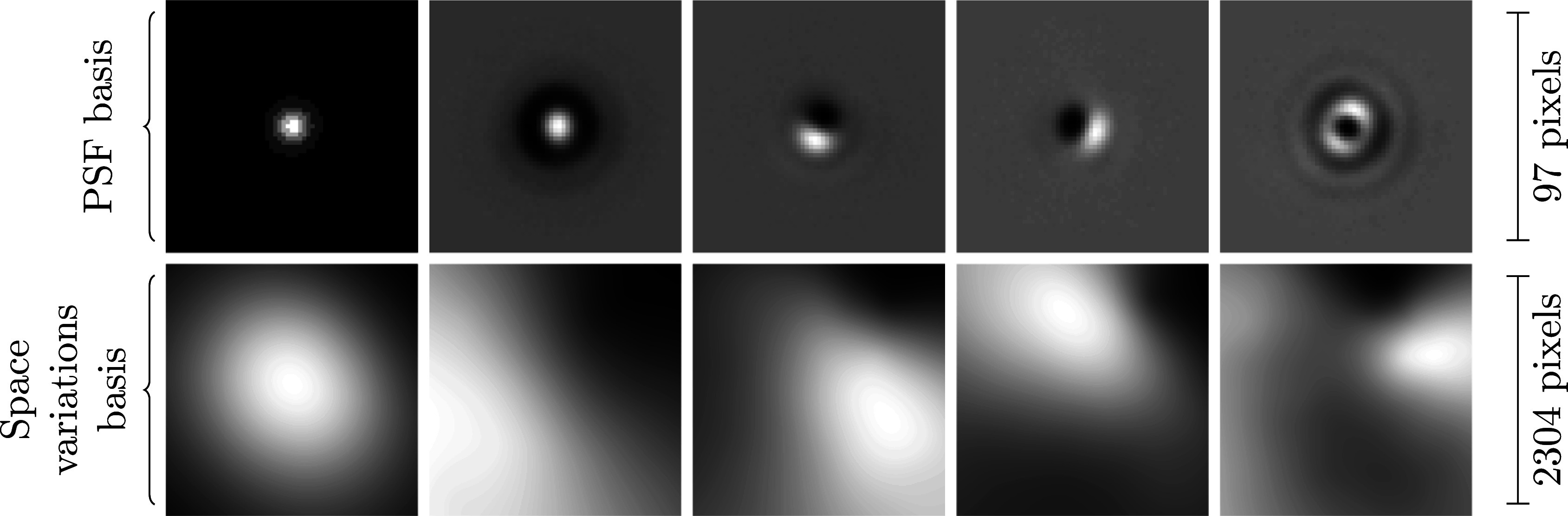
Examples of eigen-PSF and eigen-space variation bases for a wide-field microscope.^(^^28^^)^

Defining an eigen-PSF basis can be achieved by computing a principal component analysis of a family of observed or theoretical point spread functions.^(^^37^^)^ An example of an experimental eigen-PSF basis obtained in ^(^^28^^)^ is shown on Figure 2, top.

#### 2.1.3. Space-variant models and product-convolution expansions

The convolution model 2.1 can only capture *space-invariant* impulse responses. When dealing with large field of views, this model becomes inaccurate. One way to overcome this limitation is to use product-convolution expansions,^(^^28^^,^^32^^,^^33^^)^ which efficiently encode space-varying systems.

**Model 2.2** (Product-convolution expansions). *Let* 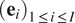 and 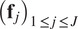 *define two orthogonal families of* 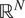 . The action of a product-convolution 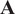 *operator reads:*
(3)

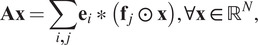

where 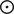 indicates the coordinate-wise (Hadamard) product.

In the above model, the basis 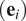 can still be interpreted as an eigen-PSF basis. Indeed, we …have for all locations 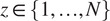 :

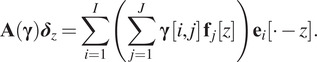



Hence, we see that each impulse response is expressed in the basis 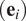 . The basis 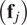 _,_ on its side, can be interpreted as an eigen-space variation basis: it describes how the point spread functions can vary in space. It can be estimated by interpolation of the coefficient of a few scattered PSF in the eigen-PSF basis 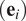 . In optical devices such as microscopes, the estimation of the families 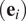 and 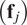 can be accomplished by observing several images of microbeads.^(^^10^^,^^28^^)^ An example of the experimental product-convolution family is shown in Figure 2 for a wide-field microscope. In that case, the dimension 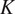 of the subspace is 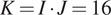 . Airy pattern oscillations are found in the first eigen-PSFs and intensity variations, such as nonhomogeneous illuminations/vignetting, in the spatial variation maps.

#### 2.1.4. Nonlinear parameterization and Zernike polynomials

An alternative to the linear models is given by the theory of diffraction. A popular and effective model in microscopy and astronomy consists of using the Fresnel/Fraunhofer theory. We can approximate the pupil function with a finite number of Zernike polynomials.^(^^40^^,^^44^^)^ This model leads to some of the state-of-the-art algorithms for blind deconvolution and superresolution in microscopy and astronomy.^(^^7^^,^^62^^,^^72^^,^^78^^)^

**Model 2.3** (Fresnel approximation and a Zernike basis). *We assume that the forward model is a convolution with a slice of a continuous 3D kernel* 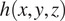 . *The 3D kernel can be expressed through the 2D pupil function* 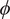 *as*


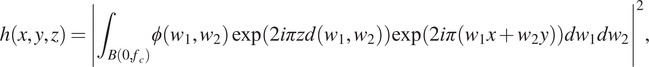

where 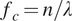 is the cutoff frequency, 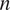 is the refractive index of the immersion medium, and 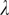 is the wavelength of the observation light and

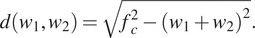

*The complex pupil function* 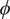 can be expanded with Zernike polynomials 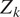 :

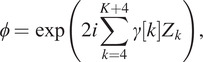


*where the coefficients* 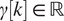 *are real number.* ^2^

**Figure 3.**
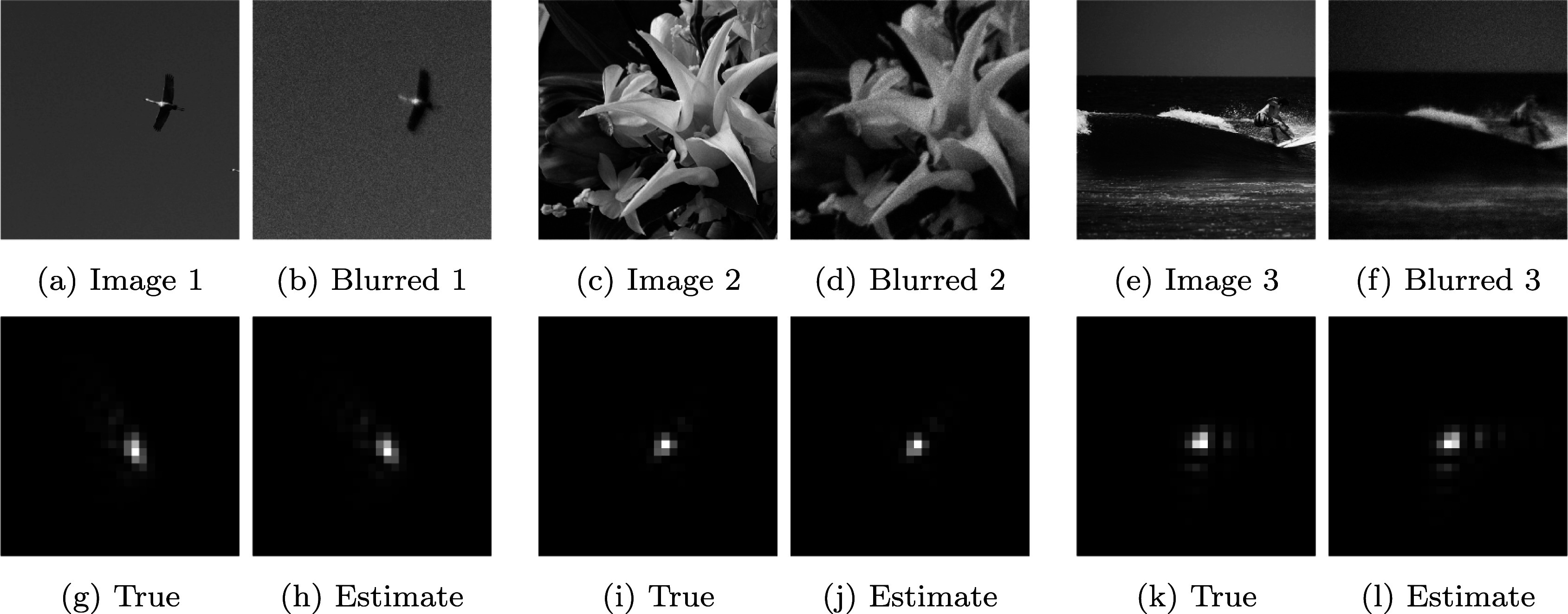
Examples of results for the identification network with convolution kernels defined through Fresnel approximation. Top: the original and blurred and noisy 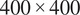 images. Bottom: the true 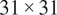 kernel used to generate the blurry image and the corresponding estimation by the neural network. Notice that there is a large amount of white Gaussian noise added to the blurred image. The image boundaries have been discarded from the estimation process to prevent the neural network from using information that would not be present in real images.

A few examples of slices of point spread functions generated with Model 2.3 are displayed in Figure 3. Notice that we do not use the first three Zernike polynomials (piston, tip, and tilt) as they do not influence the shape of the PSF. In our experiments, we used 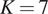 Zernike polynomials. In the Noll nomenclature, they are referred to as 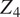 : defocus, 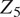 - 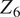 : primary astigmatism, 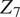 - 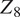 : primary coma, and 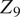 - 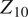 : trefoil. We set the coefficients 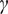 as uniform random variables with an amplitude smaller than 0.15. As can be seen, a rich variety of impulse responses can be generated with this low-dimensional model.

### 2.2. The deep-blur architecture

We propose to train two different neural networks 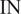 and 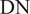 sequentially:

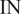
 is an *identification network.* It depends on weights 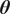 . The mapping 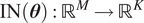 takes as an input a degraded image 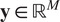 and provides an estimate 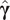 of 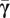 in 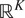 .
- DN is a *deblurring network.* It depends on weights 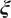 . The mapping 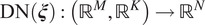 takes as input parameters the blurry image 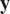 and the operator coefficient 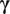 . It outputs an estimate 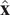 of the sharp image 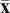 .

#### 2.2.1. The identification network

Traditional estimation of a blur kernel relies on the detection of cues in the image such as points (direct observation^(^^10^^,^^26^^,^^31^^,^^73^^)^), edges in different orientations (Radon transform of the kernel^(^^49^^)^), or textures (power spectrum^(^^38^^)^) followed by adapted inversion procedures. This whole process can be modeled by a set of linear operations (filtering) and nonlinear operations (e.g., thresholding). A convolutional neural network, composed of similar operations, should therefore be expressive enough to estimate the blur parameters. This is the case for the deep-blur identification architecture, a ResNet encoder,^(^^45^^)^ as shown in Figure 1, left. It consists of a succession of convolutions, ReLU activation, batch normalization, and average pooling layers, which sequentially reduce the image dimensions. The last layer is an adaptive average pooling layer, mapping the output of the penultimate layer to a vector of constant size 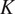 . In our experiments, the total number of trainable parameters, which includes the weights of the ResNet, that is, the convolution kernels in the convolution layers, the biases in the convolution layers and the weights of the adaptive pooling layer, is 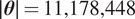 . The encoder structure has been proven to be particularly effective for a large panel of signal processing tasks.^(^^89^^)^

#### 2.2.2. The deblurring network

The proposed deblurring network mimics a Douglas–Rachford algorithm.^(^^21^^)^ It is sometimes called an unrolled or unfolded network. This type of network currently achieves near state-of-the-art performance for a wide range of inverse problems (see e.g. ^(^^59^^)^). It has the advantages of having a natural interpretation as an approximate solution of a variational problem and naturally adapts to changes of the observation operators.

*Deep unrolling.* For 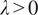 , let 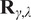 denote the following regularized inverse:

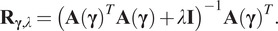

For a parameter 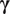 describing the forward operator and an input image 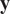 , the Douglas–Rachford algorithm can be described by the following sequence of operations, ran from 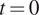 to 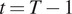 with 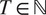 .

Algorithm 1 The Douglas–Rachford deblurring network 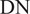 .

**Require:** iteration number 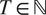 , operator 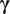 , scale 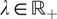

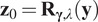

**for all** 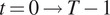 **do**

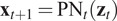

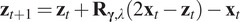

**end for**

The initial guess 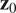 corresponds to the solution of

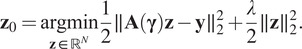



It can be evaluated approximately with a conjugate gradient algorithm run for a few iterations (20 in our implementation).

The mapping 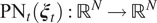 can be interpreted as a “proximal neural network.” Proximal operators^(^^21^^)^ have been used massively in the last 20 years to regularize inverse problems. A popular example is the soft-thresholding operator, which is known to promote sparse solutions. Here, we propose to learn the regularizer as a neural network denoted 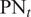 , which may change from one iteration to the next. It corresponds to the green layers in Figure 1.

The parameters 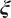 that are learned are the weights 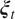 defining the 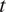 th proximal neural network 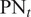 . In our experiments, the networks 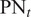 have the same architecture for all 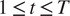 . We used the current state-of-the-art network used in plug-and-play algorithms called DRUNet.^(^^46^^,^^87^^)^ We set 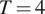 iterations. Each of the 4 proximal networks contain 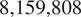 parameters, resulting in a total of 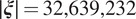 parameters to be trained.

### 2.3. Training

We propose to first train the identification network 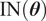 alone and then train the deblurring network 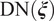 with the output of the identification network as an input parameter. This sequential approach presents two advantages:

- The memory consumption is lower. The automatic differentiation only needs to store the parameters of the individual networks, instead of both. This reduces the memory footprint.
- The identification network can be used independently of the other, and it is therefore tempting to train it separately. In metrology applications, for instance, where the aim is to follow the state of an optical system through time, the identification network IN is the most relevant brick. In some applications, such as superresolution from single molecules, the deblurring network could be replaced by a more standard total variation-based solver,^(^^13^^)^ once the operator is estimated.

In what follows, we let 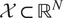 denote a dataset of admissible images/signals and 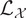 denote a sampling distribution over 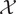 . We let 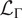 denote a sampling distribution on the set 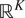 of blur parameters. In our experiments, the perturbation 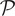 in Equation (2) is assumed to be an approximation of the Poisson-Gaussian noise.^(^^35^^)^ We assume that 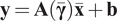 , where 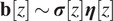 , 
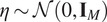
 _,_ and 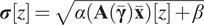 . The parameters 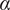 and 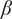 are set uniformly at random in the ranges 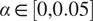 and 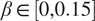 . In what follows, we let 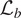 denote the noise distribution that we just described.

We propose to train both the identification and the deblurring networks using the empirical risk minimization. First, the identification network is trained by solving:(4)

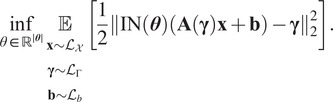



Once the identification network 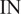 is trained, we turn to the deblurring network by solving the following optimization problem:(5)

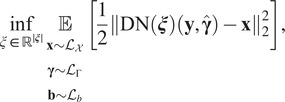

where 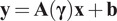 is the degraded image and 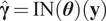 is the estimated parameter. Of importance, notice that we do not plug the true parameter 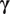 in 5, but rather the estimated one 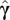 . This way, the deblurring network DN can learn to correct model mismatches that may occur at the estimation step.

The two problems above consist in constructing minimum mean square estimators (MMSE). At the end of the training procedure – under technical assumptions^(^^41^^)^ – we can consider that the networks approximate a conditional expectation:

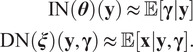



This is – by construction – the best estimators that can be generated on average. This approach is therefore really different from most alternatives in the literature, which consist of constructing MAP estimators. MMSE estimators can be expressed as integrals, which depend heavily on the operator distributions 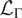 and on the image distribution 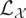 . They should therefore be constructed carefully depending on the physical knowledge of the observation system (resp. observed sample). By using the general computer vision database COCO, we hope to cover a wide range of image contents, leading to a wide-purpose method for identification. The performance could likely be improved using more specific databases. For instance, we could simulate the images according to realistic processes for specific applications such as single-molecule localization. This is out of the scope of this article. For 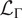 , we sample a large set of realistic parameters uniformly at random in our experiments.

The above optimization problems are solved approximately using stochastic gradient descent-type algorithms. In our experiments, we used the Adam optimizer^(^^48^^)^ with the default parameters: the learning rate is set to 0.001, betas are (0.9, 0.999), epsilon is 1e-8, weight decay is 0, and amsgrad is false.

## 3. Results

Let us illustrate the different ideas proposed in this article. In all our experiments, we trained the neural networks using the MS COCO dataset.^(^^53^^)^ It contains 118,287 images in the training set and 40,670 images in the test set. It is composed of images of everyday scenes, capturing objects in various indoor and outdoor environments. It presents substantial differences with typical microscopy images, but the high diversity and quality of the images makes it possible to construct efficient generic image priors. This was already observed in ^(^^77^^)^.

### 3.1. Convolution operators

We evaluate the accuracy of the identification and deblurring networks for convolution (i.e., space invariant) operators. We assess them for images generated with point spread functions expanded in Zernike polynomial.

#### 3.1.1. Identifying convolution operators

We assess the ability of a residual network to identify the point spread function generated by the Fresnel diffraction Model 2.3. A similar study was carried out in ^(^^76^^)^ with 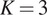 coefficients. Here, we extend the study to 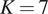 coefficients allowing us to represent the following aberrations in the Noll nomenclature^(^^63^^)^: defocus, primary astigmatism, primary coma, trefoil, and primary spherical.

We generate random PSFs by drawing the coefficients 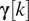 (see Model 2.3) uniformly in the range 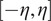 . The higher 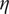 , the more spread and oscillating the PSFs. Hence, 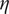 can be interpreted as a measure of PSF complexity. The model was trained for a value of 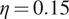 .

In the first experiment, we simply used additive white Gaussian noise (i.e. 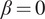 ) of standard deviation 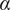 . Figure 3 shows the identification results for 3 images taken at random from the test set and 3 operators taken at random in the operator set. On these examples, the network provides faithful estimates despite a substantial noise level and images with little contents. To further characterize the network efficiency, we measure the distribution of signal-to-noise-ratio (SNR) in the noiseless regime. For a kernel 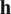 , the error of the estimated kernel 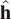 is defined by(6)

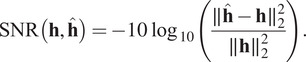



**Figure 4.**
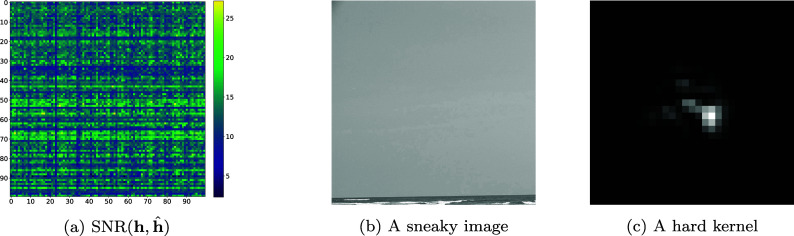
On the left: a 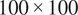 table representing the SNR of the PSF. In this table, we evaluated the identification network for 100 images (left to right) and 100 kernels (top to bottom) with no noise. As can be seen, there are horizontal and vertical stripes. This means that some images and some kernels make the identification problem easier or harder. In the middle: an image making the identification problem hard (column 23). On the right: a kernel making the identification harder (row 65).

Figure 4 summarizes the conclusions. In average, the identification network outputs estimates with a relative error below 5%.

**Figure 5.**
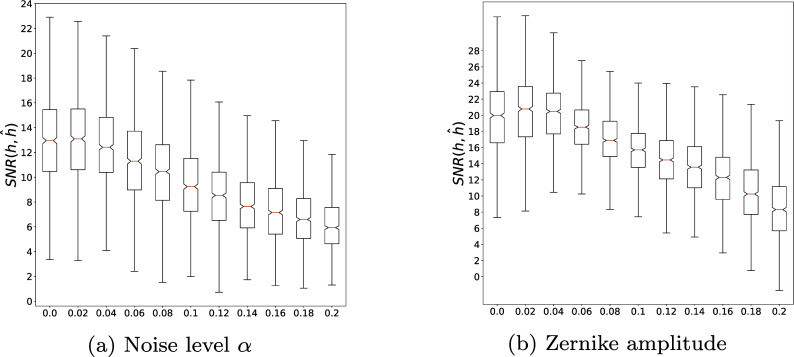
Stability of the kernel estimation with respect to noise level (left) and amplitude of the Zernike coefficients in the noiseless regime (right).

Finally, we study the stability to the noise level 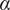 in Figure 5a – and to the PSF complexity 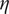 in Figure 5b. As can be seen, the identification outputs predictions with less than 10% error with a probability larger than 0.5 up to a large noise level of 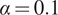 for images in the range 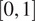 . The dependency on the kernel’s complexity, measured through the Zernike polynomials amplitude 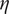 is very clear with typical errors below 2% for 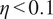 and then a relatively fast increase. It is nonetheless remarkable that the identification returns estimates with less than 15% error for 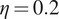 , which produces more complex PSFs than those observed during the training phase, showing some ability of the network to generalize.

#### 3.1.2. Evaluating the deblurring network

**Figure 6.**
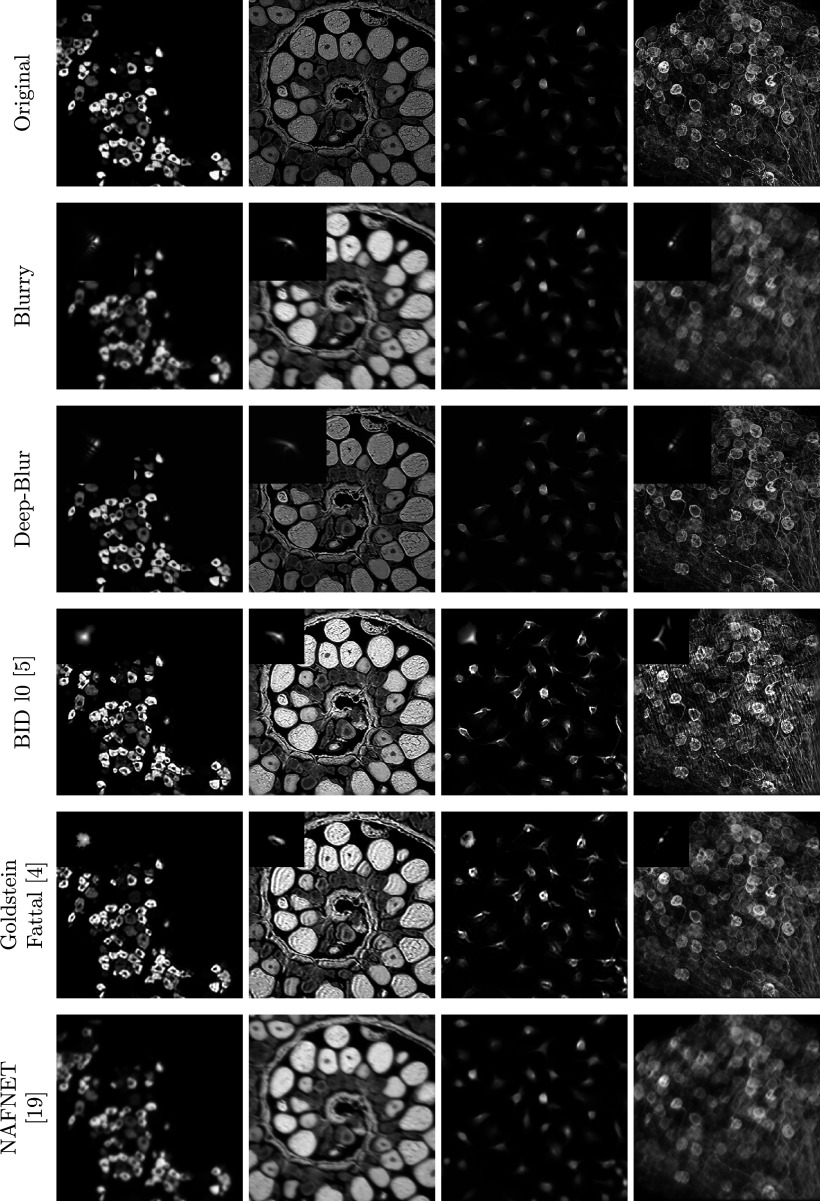
*Deep-blur in action in the noiseless setting. Quantitative evaluations are reported in Table 1. When available, the estimated blur kernel is displayed at the bottom right. First row: original images. Second row: blurry-noisy images. Third row: deep-blur. Fourth row:* ^(^^5^^)^ *Fifth row:* ^(^^4^^)^ *Sixth row:* ^(^^19^^)^.

**Figure 7.**
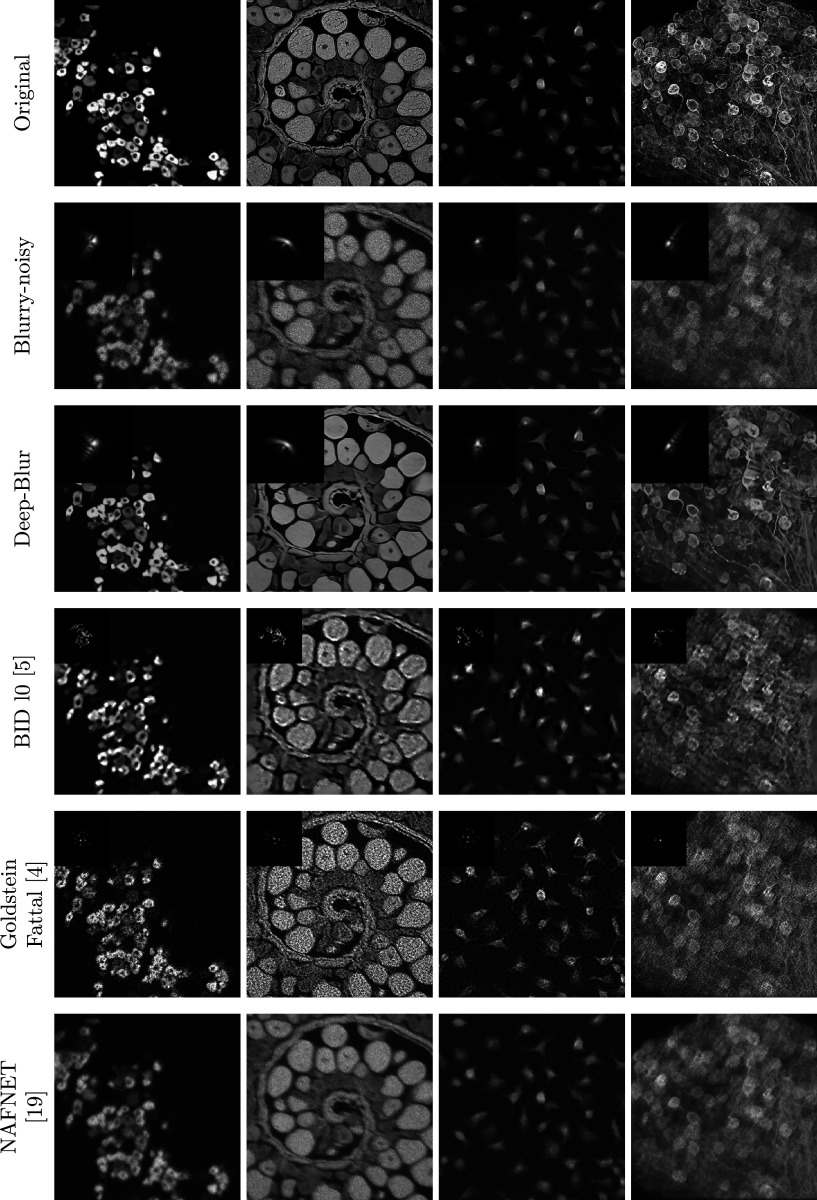
Deep-blur in action with a medium noise level ( 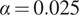 , 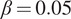 ). Quantitative evaluations are reported in Table 1. When available, the estimated blur kernel is displayed at the bottom right. First row: original images. Second row: blurry-noisy images. Third row: deep-blur. Fourth row: ^(^^5^^)^ Fifth row: ^(^^4^^)^ Sixth row: ^(^^19^^)^.

**Figure 8.**
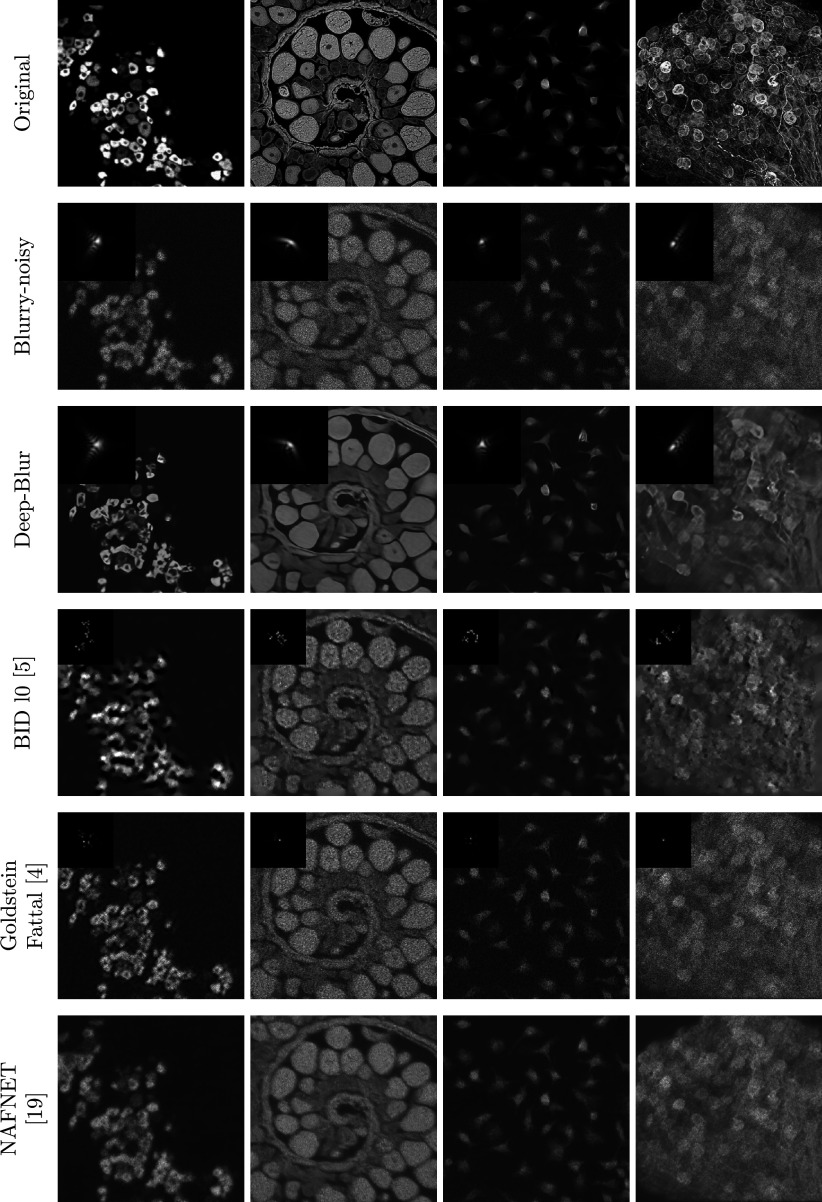
Deep-blur in action in a high-noise regime ( 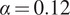 , 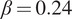 ). Quantitative evaluations are reported in Table 1. When available, the estimated blur kernel is displayed at the bottom right. First row: original images. Second row: blurry-noisy images. Third row: deep-blur. Fourth row: ^(^^5^^)^ Fifth row: ^(^^4^^)^ Sixth row: ^(^^19^^)^.

We evaluate the performance of the proposed deblurring network for convolution operators defined using the Fresnel approximation. Figures 6–8 display some deconvolution results for different methods. The corresponding image quality measures are displayed in Table 1.

**Table 1.** Reconstruction results for different noise levels and different methods

		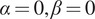	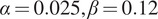	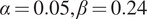
SSIM 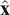	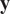	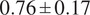	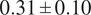	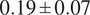
	Deep-blur	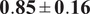	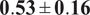	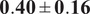
	^(^^5^^)^	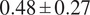	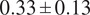	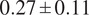
	^(^^4^^)^	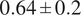	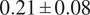	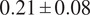
	^(^^19^^)^	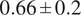	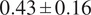	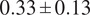
SNR 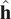	Deep-blur	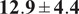	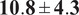	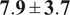
	^(^^5^^)^	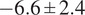	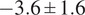	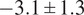
	^(^^4^^)^	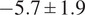	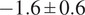	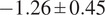

*Note*: The standard deviation is given after the symbol ±.

Bold numbers indicate the best performing method.

Notice that this problem is particularly involved: there is a complete loss of information in the high frequencies since the convolution kernels are bandlimited and we treat different noise levels up to rather high values (here 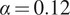 , 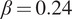 for images in the range 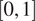 ). Despite this challenging setting, it can be seen both perceptually and from the SSIM (structural similarity index measure) that the image quality is improved whatever the noise level. It is also remarkable to observe that the proposed network architecture allows us to treat images with different noise levels. This is an important feature of the DRUNet used as a proximal network.^(^^87^^)^

We also propose some comparisons with other methods from the literature. Whenever possible, we optimized the hyperparameters by hand for each noise level to produce the best possible output. We chose the following methods:

- The 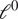 -gradient prior.^(^^66^^,^^67^^)^ This method is one of the state-of-the-art handcrafted blind deblurring methods. An efficient implementation was recently proposed in ^(^^5^^)^.
- In ^(^^38^^)^, the authors proposed a kernel estimation method based on the assumption that the image spectrum amplitude has a specific decaying distribution in the Fourier plane. The kernel estimation then boils down to a phase retrieval problem. An efficient implementation was recently proposed in ^(^^4^^)^.
- We also tested two state-of-the-art neural network approaches. The first one was a past leader of the Go-Pro deblurring challenge called NAFNET.^(^^19^^)^

The deep learning method is retrained on the same dataset as our method. As can be seen from Table 1 and the perceptual results in Figures 6–8, deep-blur outperforms the other three methods that we considered by a large margin. The PSF is recovered with an average accuracy varying between 12.9 dB in the noiseless regime to 7.9 dB in the high-noise regime using deep-blur. The image quality is improved in terms of SSIM by 0.1 in the noiseless regime to 0.2 in the high-noise regime.

**Figure 9.**
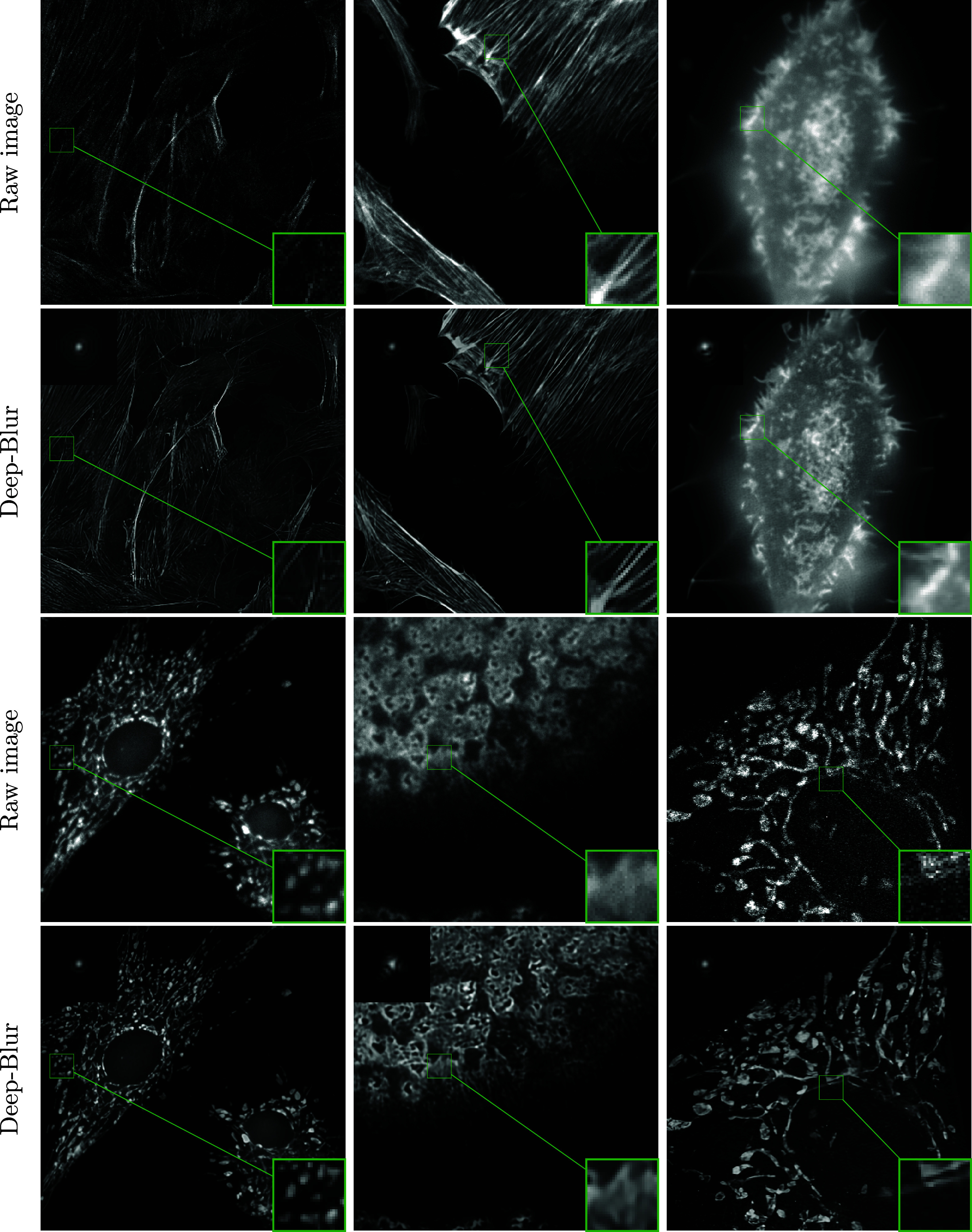
*Blind deblurring examples on real images taken from* ^(^^43^^)^*, see the samples for more details. In this experiment, only the noise level was set manually, the rest of the process is fully automatized. For this experiment, no ground truth is available and the results have to be assessed by visual inspection.*

**Figure 10.**
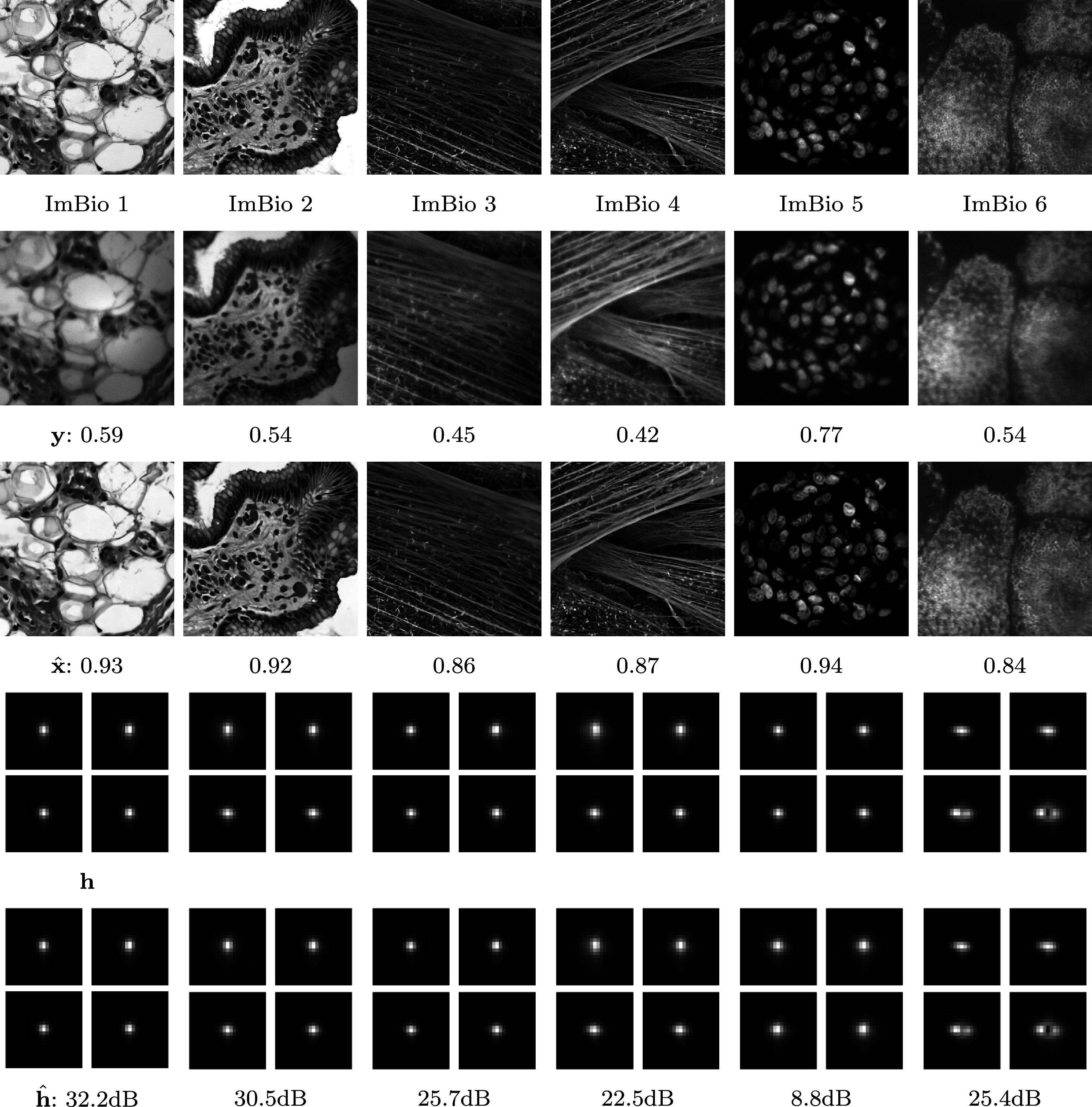
Deep-blur applied to spatially varying blur operators on microscopy images (not seen during training). The blur operators are sampled from a family estimated using a real wide-field microscope. First row: the original images. Second row: blurry-noisy images. Third row: the blind deblurring result with deep-blur. The SSIM of the resulting deblurred image is displayed below. Fourth row: The true blur operator. We display 4 evenly spaced impulse responses in the field of view. Fifth row: The estimated blur operator. The SNR of the estimated kernel is displayed in the caption in dB.

All the other methods yield negative SNR for the PSF. At a perceptual level, handcrafted methods (Goldstein-Fattal and 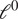 gradient prior) still recover the PSF shape approximately. The recovered image is also sharpened, but its SSIM quality is actually lowered by more than 0.1 in the noiseless regime, and improved by 0.1 in the high-noise regime. The SSIM is always lower than the one of deep-blur.

*Experiments on real images.* In Figure 9, we provide a few deep-blur results on real microscopy images from the dataset.^(^^43^^)^ We used the sample images available on the following link. As can be seen, the reconstructed images are denoised and have a better visual contrast In this experiment, we do not have a ground-truth deblurred result and the quality can only be assessed by visual inspection. Validating the estimation requires careful optics experiments, which we leave as an open topic for now.

*Training on the true or estimated operators.* At training time, we can feed the unrolled deblurring network with the operator that was used to synthesize the blurry image, or the one estimated using the identification network. The potential advantage of the second option is to train the proximal networks to correct model mismatches. We tested both solutions on two different operator families. It turns out that they led to nearly indistinguishable results overall in average. The most likely explanation for this phenomenon is that the model mismatches produced by the identification network cannot be corrected with the proximal networks.

*Memory and computing times.* The model contains about 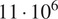 parameters for the identification part and 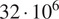 parameters for the deblurring part. This is a total of 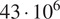 trainable parameters. This size is comparable to the usual computer vision models available in TorchVision. For example, it is slightly smaller than a ResNet101 classifier. The deep-blur model uses about 1 GB of GPU memory at test time, which can be considered lightweight, since it fits on most consumer graphics cards.

After training, it takes 0.3 seconds to identify a kernel and deblur an image of size 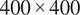 on an Nvidia RTX 8000 with 16 TFlops. For comparison, the handcrafted models used in our numerical comparisons take between 5 seconds and a few minutes to perform the same task on the CPU. No GPU implementation is provided.

### 3.2. Product-convolution operators

To finish the numerical experiments, we illustrate how the proposed ideas perform on product-convolution operators.

We first illustrate the performance of the identification network. We trained the identification network on natural images from the MS COCO dataset, but evaluate it on biological images from microscopes. We selected 6 images: *ImBio 1* is an histopathology of angiolipoma,^(^^22^^)^ *ImBio 2* is an histopathology of reactive gastropathy,^(^^23^^)^, *ImBio 3 and 4* are actin filaments within a cell,^(^^24^^)^ *ImBio 5* is an slice of a spheroid from ^(^^54^^)^, and *ImBio 6* is a crop of a podosome obtain on a wide-field microscope.^(^^12^^)^

The blur operators are generated by Model 2.2 using 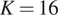 parameters. The blur model is obtained following the procedure described in ^(^^28^^)^. To compute the product-convolution decomposition described in Model 2.2, we collected 18 stacks of 21 images of microbeads spaced by 200 nm on a wide-field microscope with a ×100 objective lens (CFI SR APO 100XH NA 1,49 DT 0,12 Nikon) mounted on a Nikon Eclipse Ti-E and a Hamamatsu sCMOS camera (ORCA FLASH4.0 LT). Figure 10 shows the identification results. The blur coefficients predicted by the deep-blur identification are accurate estimates in all cases. On average, the SNR is much higher than in the previous experiment, which can likely be explained by a smaller dimensionality of the operators’ family. In all cases, the image quality is improved despite an additive white Gaussian noise with 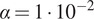 and 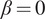 . This is remarkable since this type of image is different from the typical computer vision images found in the MS COCO dataset.

## 4. Discussion

We proposed an efficient and lightweight network architecture for solving challenging blind deblurring problems in optics. An encoder first identifies a low-dimensional parameterization of the optical system from the blurry image. A second network with an unrolled architecture exploits this information to efficiently deblur the image. The performance of the overall architecture compares favorably with alternative approaches designed in the field of computer vision. The principal reason is that our network is trained using fine physical models obtained using Fresnel diffraction theory or experimental data providing accurate space-varying models. A second reason is that the unrolled architecture proposed herein emerges as a state-of-the-art competitor for a wide range of inverse problems. Overall, we believe that the proposed network, trained carefully on a large collection of blurs and images could provide a universal tool to deblur microscope images. In the future, we would like to carry out specific optical experiments to ensure that the results obtained with synthetic data are reproducible and trustworthy with real images. The initial results, obtained without reference images for comparisons are however really encouraging.

*Differences with plug and play and deep unrolling.* The proposed unrolled architecture follows closely the usual unrolled algorithms.^(^^1^^,^^2^^,^^51^^,^^52^^,^^59^^)^ There is, however, a major difference: traditionally, these unrolled architectures are trained to invert a *single operator.* In this article, we train the network with a *family* of operators. The results we obtained confirm some results obtained in ^(^^41^^)^: this approach only results in marginal performance loss for a given operator if the family is sufficiently small while providing a massive improvement in adaptivity to all the operators. In a sense, the proposed approach can be seen as an intermediate step between the plug-and-play priors (related to diffusion models^(^^42^^)^), which are designed to adapt to all possible operators, and the traditional unrolled algorithm is adapted to a single one.

*The limits of a low-dimensional parameterization.* The models considered are sufficiently rich to describe most optical devices accurately. In microscopy, they can capture defocus, refractive index mismatches, changes of temperature, tilts of optical components, usual optical aberrations with a parameter dimension 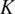 smaller than 20.

Notice however that some phenomena can hardly be modeled by low-dimensional parameterization. In microscopy, for instance, diffraction by the sample itself can lead to extremely complicated and diverse forward models better described by nonlinear equations (see e.g., ^(^^71^^)^). Similarly, in computer vision, motion and defocus blurs can vary abruptly with the movement and depth of the objects. The resulting operators would likely require a large number of parameters, which is out of the scope of this article.

Overall, we see that the proposed contribution is well adapted to the correction of systems with slowly varying point spread functions but probably does not extend easily to fast variations that can be induced by some complex biological samples.

## 5. Conclusion

We proposed a specific neural network architecture to solve blind deblurring problems where distortions come from the optical elements. We evaluated its performance carefully on blind deblurring problems with space invariant and space-varying operators. A key assumption is to have access to a forward model that depends on a set of parameters. The network first estimates the unknown parameters describing the forward model from the measurements with a ResNet architecture. In a second step, an unrolled algorithm solves the inverse problem with a forward model that was estimated at the previous step. After designing a careful training procedure, we showed an advantage of the proposed approach in terms of robustness to noise levels and adaptivity to a vast family of operators and conditions not seen during the training phase.

## Data Availability

The deep-blur architecture and the trained weights can be provided on explicit demand by e-mail at pierre.weiss@cnrs.fr.

## Footnotes

2 In this model, only the phase of the pupil function varies. In all generality, the amplitude could vary as well at a slower rate. Most models in the literature assume a constant amplitude.
